# PACAP27 mitigates an age‐dependent hippocampal vulnerability to PGJ2‐induced spatial learning deficits and neuroinflammation in mice

**DOI:** 10.1002/brb3.1465

**Published:** 2019-11-25

**Authors:** Jorge A. Avila, Magdalena Kiprowska, Teneka Jean‐Louis, Patricia Rockwell, Maria E. Figueiredo‐Pereira, Peter A. Serrano

**Affiliations:** ^1^ Department of Psychology Hunter College City University of New York New York NY USA; ^2^ The Graduate Center of CUNY New York NY USA; ^3^ Department of Biological Sciences Hunter College City University of New York New York NY USA

**Keywords:** aging, CA1, CA3, Fluoro‐Jade C, microglia, radial arm maze

## Abstract

**Background:**

Inflammation in the brain is mediated by the cyclooxygenase pathway, which leads to the production of prostaglandins. Prostaglandin (PG) D2, the most abundant PG in the brain, increases under pathological conditions and is spontaneously metabolized to PGJ2. PGJ2 is highly neurotoxic, with the potential to transition neuroinflammation into a chronic state and contribute to neurodegeneration as seen in many neurological diseases. Conversely, PACAP27 is a lipophilic peptide that raises intracellular cAMP and is an anti‐inflammatory agent. The aim of our study was to investigate the therapeutic potential of PACAP27 to counter the behavioral and neurotoxic effects of PGJ2 observed in aged subjects.

**Methods:**

PGJ2 was injected bilaterally into the hippocampal CA1 region of 53‐week‐old and 12‐week‐old C57BL/6N male mice, once per week over 3 weeks (three total infusions) and included co‐infusions of PACAP27 within respective treatment groups. Our behavioral assessments looked at spatial learning and memory performance on the 8‐arm radial maze, followed by histological analyses of fixed hippocampal tissue using Fluoro‐Jade C and fluorescent immunohistochemistry focused on IBA‐1 microglia.

**Results:**

Aged mice treated with PGJ2 exhibited spatial learning and long‐term memory deficits, as well as neurodegeneration in CA3 pyramidal neurons. Aged mice that received co‐infusions of PACAP27 exhibited remediated learning and memory performance and decreased neurodegeneration in CA3 pyramidal neurons. Moreover, microglial activation in the CA3 region was also reduced in aged mice cotreated with PACAP27.

**Conclusions:**

Our data show that PGJ2 can produce a retrograde spread of damage not observed in PGJ2‐treated young mice, leading to age‐dependent neurodegeneration of hippocampal neurons producing learning and memory deficits. PACAP27 can remediate the behavioral and neurodegenerative effects that PGJ2 produces in aged subjects. Targeting specific neurotoxic prostaglandins, such as PGJ2, offers great promise as a new therapeutic strategy downstream of cyclooxygenases, to combat the neuronal deficits induced by chronic inflammation.

## Graphical abstract

PGJ2 was infused into the hippocampus of young and aged adolescent mice. Learning and memory assessments on the radial arm maze revealed that only aged mice exhibited cognitive deficits following infusions. This indicates that aged mice are susceptible to neuroinflammation propagated by PGJ2.

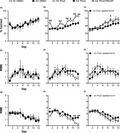

## 1 INTRODUCTION

Neuroinflammation is a defense process activated upon CNS injury to initiate repair mechanisms acutely, while chronic neuroinflammation can exacerbate, spread, and prolong CNS injury. Chronic neuroinflammation is implicated in a variety of neurological and neurodegenerative disorders, including Alzheimer's disease (AD) (Glass, Saijo, Winner, Marchetto, & Gage, 2010; Herrup, 2010; Liu & Hong, 2003). The principle mediators of CNS neuroinflammation are prostaglandins (PGs) (Iadecola & Gorelick, 2005) that are produced from arachidonic acid via the cyclooxygenase pathway (Cudaback, Jorstad, Yang, Montine, & Keene, 2014). We focused our studies on PGJ2 as it is highly neurotoxic compared to PGD2 and PGE2 (Li, Jansen, et al., 2004; Li, Melandri, et al., 2004) and can lead to neuronal dysfunction (Figueiredo‐Pereira, Rockwell, Schmidt‐Glenewinkel, & Serrano, 2015). PGJ2 is a product of spontaneous nonenzymatic dehydration of PGD2, which is the most abundant prostaglandin in the brain (Uchida & Shibata, 2007), and its levels change the most under pathological conditions (Liang, Wu, Hand, & Andreasson, 2005). Specifically, cortical neurons in AD brains were shown to exhibit accelerated PGD2 synthesis (Iwamoto, Kobayashi, & Kosaka, 1989; Yagami, 2006). PGD2 is unstable and is rapidly converted to PGJ2 (Suzuki, Hayashi, & Hayaishi, 1986). In rodents, PGJ2 levels increase upon traumatic brain injury (TBI) (Hickey et al., 2007) and stroke (Liu et al., 2011, 2013), to levels shown to be neurotoxic in vitro (Kunz, Marklund, Hillered, & Oliw, 2002). In addition, PGJ2 is taken up by cells via a carrier‐mediated active transport, ending up in the cytoplasm and nucleus (Narumiya & Fukushima, 1986).

PGJ2 signals in part via one of the PGD2 receptors, that is, the DP2 receptor, which is expressed in the cerebral cortex and hippocampus (Hata, Zent, Breyer, & Breyer, 2003; Liang et al., 2005; Monneret, Li, Vasilescu, Rokach, & Powell, 2002). DP2 is coupled to inhibitory G‐proteins, and thus, its activation lowers cAMP and increases calcium levels (Chen et al., 2013; Metcalfe, Huang, & Figueiredo‐Pereira, 2012). In an effort to overcome the cAMP deficit induced by PGJ2, we showed that raising intracellular cAMP levels with PACAP27 overcomes some of the neurotoxic effects of PGJ2 in rat cerebral cortical neuronal cultures (Metcalfe et al., 2012), and in mice exhibiting PGJ2‐induced parkinsonian‐like pathology (Shivers, Nikolopoulou, Machlovi, Vallabhajosula, & Figueiredo‐Pereira, 2014). PACAP27 is a lipophilic peptide that binds to the seven transmembrane G‐coupled receptor PAC1R (pituitary adenylate cyclase 1 receptor) at nanomolar levels, activating adenylate cyclase and elevating cAMP (Moody, Ito, Osefo, & Jensen, 2011). PAC1R is expressed in the cerebral cortex and hippocampus as well as other brain areas (Joo et al., 2004). Thus, PACAP27 may offer a novel therapeutic approach to target neurodegenerative processes because it not only ameliorates some of the pathology observed in disease models, but it also diminishes some of the clinical symptoms associated with AD and Parkinson's disease (PD) (Yang et al., 2015).

Based on the vulnerability of the hippocampus to age‐dependent cognitive decline (Driscoll & Sutherland, 2005; Rosenzweig & Barnes, 2003; Stephens, Quintero, Pomerleau, Huettl, & Gerhardt, 2011) and neurodegenerative‐associated pathogenesis, we investigated the effects of the neurotoxic PGJ2 on hippocampal‐dependent learning and long‐term memory retrieval across two distinct ages, young adult (YA, 12 weeks old) and aged adult (AA, 53 weeks old) mice. Neurodegeneration was evaluated with Fluoro‐Jade C (FJC) staining, and microglia activation with Iba‐1 staining. While acute activation of microglia is intrinsic to immune function (Schwab & Schluesener, 2004), overactivation of microglia associated with chronic inflammation is known to contribute to neuronal damage, particularly in neurodegenerative diseases (Doi et al., 2009) and memory deficits (Hou et al., 2014; Morris, Clark, Zinn, & Vissel, 2013; Smith, Yao, Chen, & Kirby, 2018). Thus, inhibition of microglial activation and the subsequent inflammatory process is regarded as an important therapeutic target for numerous neurodegenerative diseases (Hensley, 2010). We investigated here the potential benefits of PACAP27, since PACAP has been shown to be a potent regulator of the microglial response to stroke (Brifault et al., 2015) and TBI, reducing neuronal death (Mao et al., 2012).

Our findings indicate that PGJ2 can initiate age‐dependent neurodegeneration as well as its retrograde spread within the hippocampus, mimicking the cognitive and histological pathology that is relevant to AD. Furthermore, PACAP27 mitigates the spatial learning and long‐term memory deficits induced by PGJ2. Our results provide an experimental strategy to test neuroprotective therapeutics like PACAP27, that are applicable to AD and other neurodegenerative disorders in which the cyclooxygenase pathway of inflammation is involved.

## 2 MATERIALS AND METHODS

Our study followed the strict recommendations in the Guide for the Care and Use of Laboratory Animals of the National Institutes of Health (NIH). Our protocol was approved by the Hunter College, CUNY Institutional Animal Care and Use Committee. Surgeries were carried out under isoflurane anesthesia, and we made all efforts to minimize animal suffering.

### 2.1 Animals

**Figure 1:**
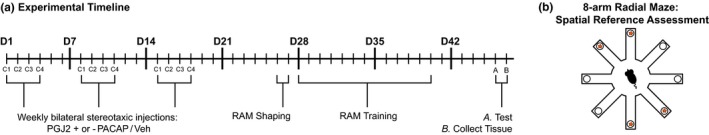
Experimental design and timeline. (a) Groups of aged adults (AA, 53 weeks old) and young adults (YA, 12 weeks old) were injected bilaterally once per week for 3 weeks with PGJ2, DMSO, or PGJ2 + PACAP27 into hippocampal CA1. Mice were grouped into drug‐balanced cohorts (C1‐C4) and injected on the same day every week. Following injections, mice were placed on rest for 7–10 days. On day 26, 8–11 days after their last injection, mice started 2 days of radial arm maze (RAM) shaping followed by RAM training for 12 days. (b) RAM training consisted of four baited and four unbaited arms in a unique pattern assigned to each animal that remained consistent for all training days. After 12 days of RAM training (six consecutive trials per day), mice received no RAM training for 6 days and then tested for RAM memory retention. Tissue was collected the following day

Male C57BL/6N mice were purchased from Taconic Farms, at two distinct ages, 12 weeks of age representing young adults (YA) and 53 weeks of age representing aged adults (AA). Mice were single housed on a 12‐hr light/dark cycle with food and water available ad libitum and were habituated for one week before treatment. Mice were randomly assigned to the treatment groups (Figure 1a). DMSO/PBS vehicle treated included AA (*n* = 7) and YA (*n* = 3). PGJ2 treated included AA (*n* = 7) and YA (*n* = 5). To assess the therapeutic efficacy of PACAP27, one group of AA were treated with PGJ2 + PACAP27 (*n* = 6). Because of the neuroprotective effects of estrogen in AD models (Merlo, Spampinato, & Sortino, 2017), our study focused only on male mice to avoid the confounding effects of the estrous cycle. Future studies will examine the cognitive and neurodegenerative effects of PGJ2 on female rodents.

### 2.2 Drugs

Commercially available PGJ2 (cat. # 18,500, Cayman Chemical) was resuspended in DMSO, and PACAP27 (pituitary adenylate cyclase‐activating polypeptide, cat. # H‐1172, Bachem Bioscience) was resuspended in sterile water. The final DMSO concentration in PBS was 17% for all microinfusions. The solutions were freshly prepared and stored for a maximum of 2 hr at 4°C and in the dark.

### 2.3 CA1 hippocampal microinfusions

We followed the same procedures as described in our previous study (Pierre, Lemmens, & Figueiredo‐Pereira, 2009), except that mice received bilateral injections targeting the hippocampal CA1 subregion. Injections were administered 1x per week, across 3 weeks, resulting in a total of 3 injections (see Figure 1). The 2 μl injections contained either vehicle (17% DMSO/injection), PGJ2 (16.7 µg/injection), or PGJ2 + PACAP27 (16.7 µg + 50 ng (respectively)/injection). For stereotaxic microinfusions, mice were anesthetized by isoflurane inhalation (induction 2%–2.5%, maintenance 1.5%–2%) administered in 100% oxygen and placed into a stereotaxic frame (Model 51730D, Stoelting Co.) fitted with a gas anesthesia mask (Model 50264, Stoelting Co.). A burr hole was drilled in the skull at coordinates relative to bregma for the hippocampal CA1 subregion: rostral–caudal −2.0 mm, medial–lateral ± 1.0 mm, and dorsal–ventral + 1.5 mm, as specified in the mouse brain atlas (Paxinos & Franklin, 2001). A 2‐µl microinjection Hamilton syringe (7,002 KH) with a 25‐gauge needle was slowly inserted into the brain and left in place for 5 min. Thereafter, 2 µl of solution was infused at an injection rate of 0.2 μl/min (Quintessential stereotaxic injector, Model 53311, Stoelting Co.). The needle was left in place an additional 5 min to ensure total diffusion of the solution. Following injection, the needle was slowly removed, and the incision was closed with monofilament absorbable sutures (cat. # 033899; Butler Schein Animal Health, Dublin, OH). After surgery, mice were administered a subcutaneous injection of 0.5 cc Lactated Ringer's solution, given wet palatable rodent chow, and kept in a warm place to recover. Subsequent injections were administered via the same drill hole established during the first surgical procedure.

### 2.4 Radial 8‐arm maze memory assessment

RAM is a behavioral task used to evaluate long‐term reference and short‐term working memory simultaneously (Sebastian, Diallo, Ling, & Serrano, 2013; Sebastian, Vergel, Baig, Schrott, & Serrano, 2013; Serrano et al., 2008). 8–11 days after their last injection, mice began RAM shaping. Briefly, mice were food restricted to 85% of free‐feeding weight. To acclimate mice to the maze prior to training, each mouse received three exposures/day (2 days) to the maze with all arms baited (10‐min intervals, followed by 1 hr in home cage). During training, four of the 8 arms were baited with wet sweetened oatmeal (Maypo Inc, NJ) as previously described (Sebastian, Vergel, et al., 2013; Sebastian, Vergel, et al., 2013; Serrano et al., 2008). The sequence of baited arms remained fixed for each mouse. To avoid the use of internal maze cues, each day the maze was rotated 90° keeping the position of the baited arms stationary with respect to the room cues. Mice received six consecutive trials per day lasting no more than 3 min per trial. To establish the trial % correct score, we divided the number of food rewards collected by the total number of arms entered. Reference and working memory errors were also scored. Reference memory errors occur when a subject entered an arm that is never baited. Reference memory is also associated with long‐term memory performance. Working memory errors were committed when a subject re‐entered an arm where the food reward had already been collected for that trial.

### 2.5 Fluoro‐Jade C staining

Following behavioral analyses, mice were terminally anesthetized (i.p.) with ketamine (100 mg/kg) and acepromazine (3 mg/kg), and transcardially perfused with PBS followed by 4% paraformaldehyde in PBS. The mouse brains were removed, postfixed overnight at 4°C, followed by cryoprotection (30% sucrose/PBS at 4°C). Brains were sectioned in the coronal plane using a freezing microtome at a thickness of 20 μm, and sections were collected serially along the rostrocaudal axis of the hippocampus. Tissue series were stored at −20°C (cryoprotectant: 30% glycerol and 30% ethylene glycol in PBS) until used. Visual confirmation of injection track or similar cortical damage was noted for each brain during sectioning to be near targeted coordinates: rostral‐caudal −2.0 mm, medial‐lateral ± 1.0 mm, dorsal‐ventral +1.5 mm relative to Bregma, corresponding to figures 44–47 of the mouse brain atlas (Paxinos & Franklin, 2001). Degenerating neurons/terminals were detected with Fluoro‐Jade C (cat# AG325, Millipore) as described in Damjanac et al. (2007); Schmued, Stowers, Scallet, and Xu (2005). Sections were processed with a mounted protocol. Slides containing hippocampal sections were immersed in 0.06% KMnO4 for 10 min rinsed 2x in dd‐H_2_O, immersed in 0.001% Fluoro‐Jade C for 5 min, immersed in 10% glacial acetic acid for 10 min, and rinsed 2x in dd‐H_2_O before cover‐slipping with DPX mounting media, adapted from previously described work (Schmued et al., 2005). Slides were kept at room temperature in the dark until imaged.

### 2.6 Immunohistochemistry

A second set of hippocampal brain sections cut at 20 µm were processed for Iba‐1 (Cat. # 019‐19741 WAKO Chem.) and NeuN (Abcam prod. # 134014) immunofluorescent staining. Sections were processed with a mounted protocol. After mounting, hippocampal sections were immersed in 0.05 M glycine in 0.3% Triton in PBS (T‐PBS) for 30 min to reduce autofluorescence, followed by rinses in 0.3% T‐PBS, followed by incubation in 15% bovine serum albumin (BSA) in 0.3% T‐PBS blocker, and finally primary antibody cocktail containing 15% BSA and 0.03% T‐PBS overnight at 4°C on a rocker. Primary antibodies were diluted at 1:500 concentration in solution. The following day, sections were washed in 0.03% T‐PBS, incubated in fluorescent secondary antibody cocktail 15% BSA and 0.03% T‐PBS for 2 hr. Secondary antibodies included Alexa Fluor goat anti‐chicken 568 (cat# A‐11041, Life Technologies, Thermo Fisher Sci.) and Alexa Fluor goat anti‐rabbit 488 (cat# A‐11008, Life Technologies, Thermo Fisher Sci.) at a 1:250 concentration in solution. Sections were washed with 0.03% T‐PBS, then PBS, and finally were cover‐slipped with VectaShield^®^ mounting media. Slides were kept at 4°C in the dark until imaged.

### 2.7 Immunohistochemistry and Fluoro‐Jade C quantification

Under a wide‐field fluorescence microscope, Zeiss Axio Imager, the software AxioVision was used to capture whole hippocampal region mosaics via MosaiX capture mode at 20x magnification. Exposure times for respective channels were kept constant between sections. GFP spectrum filter set was used for Fluoro‐Jade C and Iba‐1 imaging and DSRed set was used for NeuN. For each captured image, ZVI files were loaded onto Image J (NIH). Hippocampal subfields were isolated, cropped, and saved as.tif files for use in pixel‐intensity area analyses. Images were analyzed to extract the positive signal from each image with custom batch‐processing macroscripts created for each channel/marker. Pixel‐intensity statistics were calculated from the images at 16‐bit intensity bins. Positive signal within each cropped image was extracted using the following formulae: average pixel intensity + [(1.5 *[NeuN]* or 2.5 *[Iba‐1]* or 2.0 *[Fluoro‐Jade C]*) x Standard deviation of intensities] (Kerfoot, Agarwal, Lee, & Holland, 2007). Positive signal areas were measured, and masks were created and merged when colocalization analyses followed. Colocalization measurements were achieved by measuring overlap of two merged masks from an analyzed image crop. IBA‐1 images were also used for morphological analyses, to characterize microglia into 3 distinct shape classes based on the form factor (FF) value derived from Image J's circularity function: 4π * area/perimeter^2^. Microglia within each cropped IBA‐1 image were extracted using the following formula: average pixel intensity + [2 x Standard deviation of intensities], and particles within 50–300 microns^2^ were chosen for FF analyses. The three microglial classes were determined as follows: ramified morphology = FF of 0.10–0.4; reactive morphology = FF of 0.45–0.70; and amoeboid morphology = FF of 0.75–1.00 (Figure 6d). Quantities of each microglia class were collected and analyzed in ratios to each other, and then compared across treatments. Fluoro‐Jade C and IBA‐1/NeuN data are presented as % change from DMSO controls (YA + AA). For Fluoro‐Jade C analysis, one (1) unilateral section per animal was used for quantification unless imaging revealed significant damage to sections, in which case a second adjacent section was stained and imaged. For IBA‐1/NeuN analysis, one (1) unilateral section per animal was used for quantification as well. Sections chosen for analysis corresponded to figures 43–47 of the mouse brain atlas (Paxinos & Franklin, 2001), spanning approximately 0.4 mm with an approximate range of distance of 0.1–0.6 mm away from the targeted injection site. Contralateral brain hemispheres were used for a separate study.

### 2.8 Statistics

**Figure 2:**
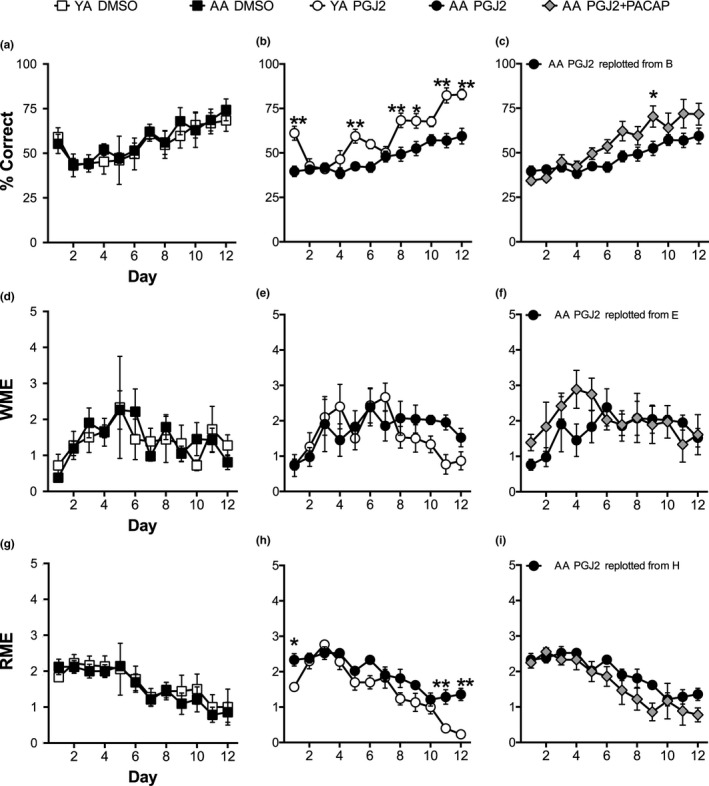
PGJ2 induces spatial learning deficits in AA that is mitigated by PACAP27. (a–c) % correct scores for DMSO‐treated AA and YA groups (a) show equivalent learning over training days (*p* < .01) with no effect on age. (b) PGJ2‐treated AA and YA show an effect of training (*p* < .01), age (*p* < .01), and an interaction (*p* < .01). Post hoc, training days 1, 5, 8, 9, 11, and 12 are significant (*p < .05, **p < .01). (c) AA treated with PGJ2 + PACAP27 versus PGJ2 show an effect of training (*p* < .01) and drug treatment (*p* < .05). Post hoc, training day 9 is significant (*p < .05). (d–f) Working memory errors (WME) show no effect on age or drug treatment for AA and YA treated with (d) DMSO, (e) PGJ2, and (f) PGJ2 + PACAP27 versus PGJ2 in AA. (g–i) Reference memory errors (RME) for (g) DMSO‐treated AA and YA groups show an effect of training (*p* < .01) with no effect on age. (h) PGJ2‐treated AA and YA show an effect of training (*p* < .01), age (*p* < .01), and an interaction (*p* < .01). Post hoc, training days 1, 11, and 12 are significant (*p < .05, **p < .01). (i) AA treated with PGJ2 + PACAP27 versus PGJ2 show an effect of training (*p* < .01) but no effect of drug treatment

**Figure 3:**
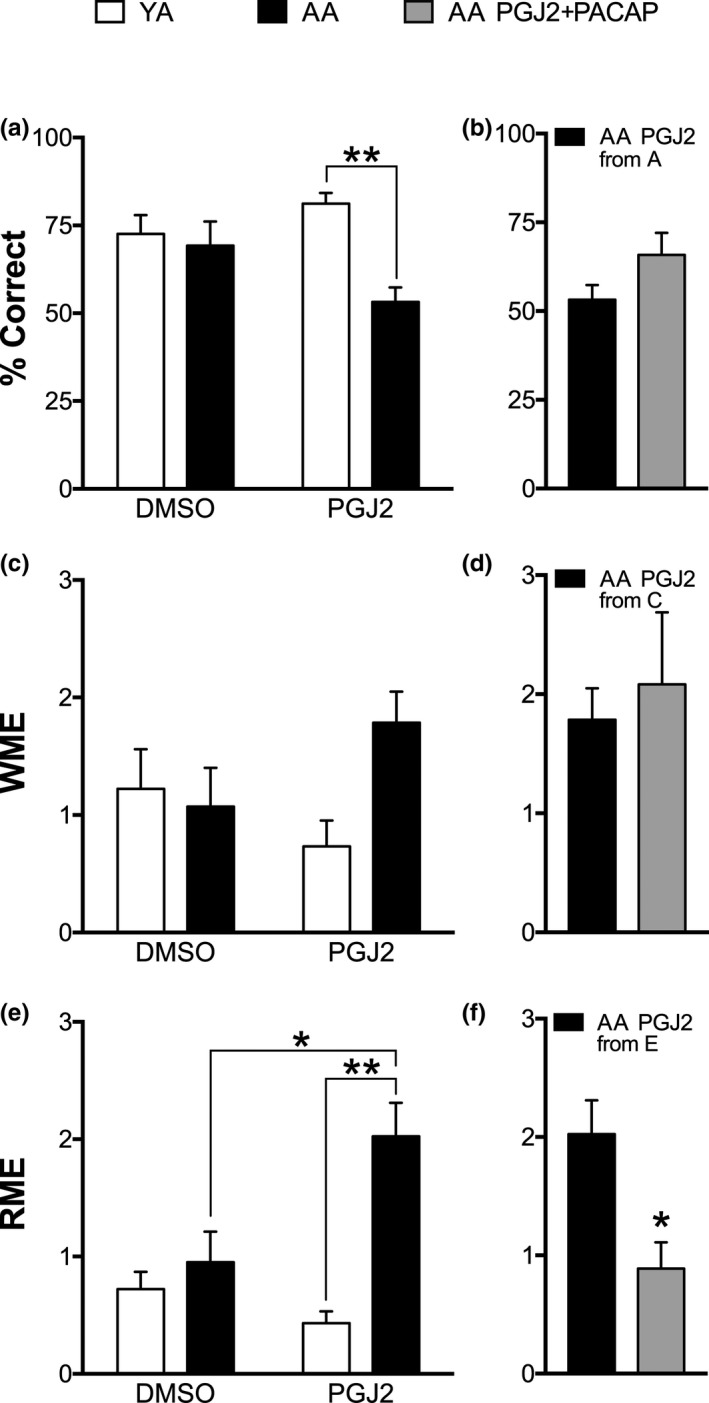
PGJ2 disrupts long‐term memory retention in AA that is mitigated by PACAP27. Six days following the last RAM training day, mice were given an additional 6 RAM trials. (a) % correct scores for DMSO versus PGJ2 treated in AA and YA groups show an effect of age (*p* < .05). Post hoc, PGJ2 treated AA versus YA is significant (**p < .01). (b) % correct scores for AA treated with PGJ2 + PACAP27 versus PGJ2 is not significantly different. (c) Working memory errors (WME; average/6 trials) for DMSO versus PGJ2 treated in AA and YA groups show no effect of drug or age. (d) WME for AA treated with PGJ2 + PACAP27 versus PGJ2 are not significantly different (*p* > .05). (e) Reference memory errors (RME; average/6 trials;) for DMSO versus PGJ2 treated in AA and YA groups show an effect of age (*p* < .01) and an interaction (*p* < .05). Post hoc, PGJ2 treated AA versus YA (**p < .01) and AA treated with PGJ2 versus DMSO (*p < .05) are significant. (f) RME for AA treated with PGJ2 + PACAP27 versus PGJ2 is significant (*p < .05)

**Figure 4:**
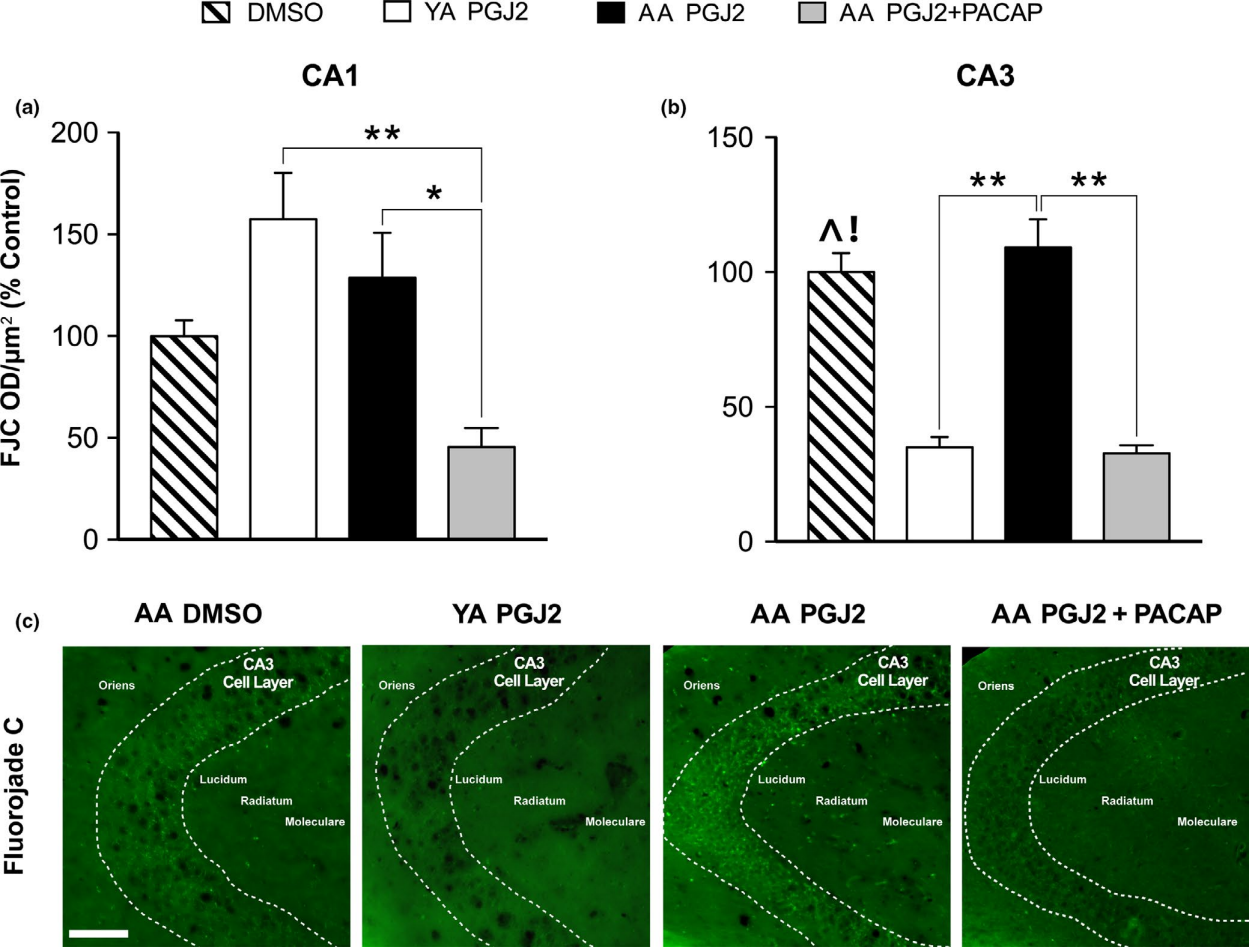
PGJ2‐treated AA show increased hippocampal neural degeneration in CA3 pyramidal cells that is mitigated by PACAP27. (a) In CA1, FJC shows an effect of treatment (*p* < .01). Post hoc, AA treated PGJ2 + PACAP27 versus AA PGJ2 (*g = 1.63, *p* < .05) and versus YA PGJ2 (**g = 2.61, *p* < .01) are significant. (b) In CA3, FJC shows an effect of treatment (*p* < .01). Post hoc, PGJ2‐treated AA versus YA (**g = 3.09, *p* < .01) and AA treated PGJ2 + PACAP27 versus PGJ2 (**g = 3.23, *p* < .01) are significant. Additionally, significant differences were found between DMSO and YA PGJ2 (^*g* = 3.17, *p* < .001) as well as DMSO and AA PGJ2 + PACAP (!*g* = 3.285, *p* < .001). (c) FJC staining represented in CA3 across conditions. Stippled area shows CA3 cell layer; bar = 100 µm

**Figure 5:**
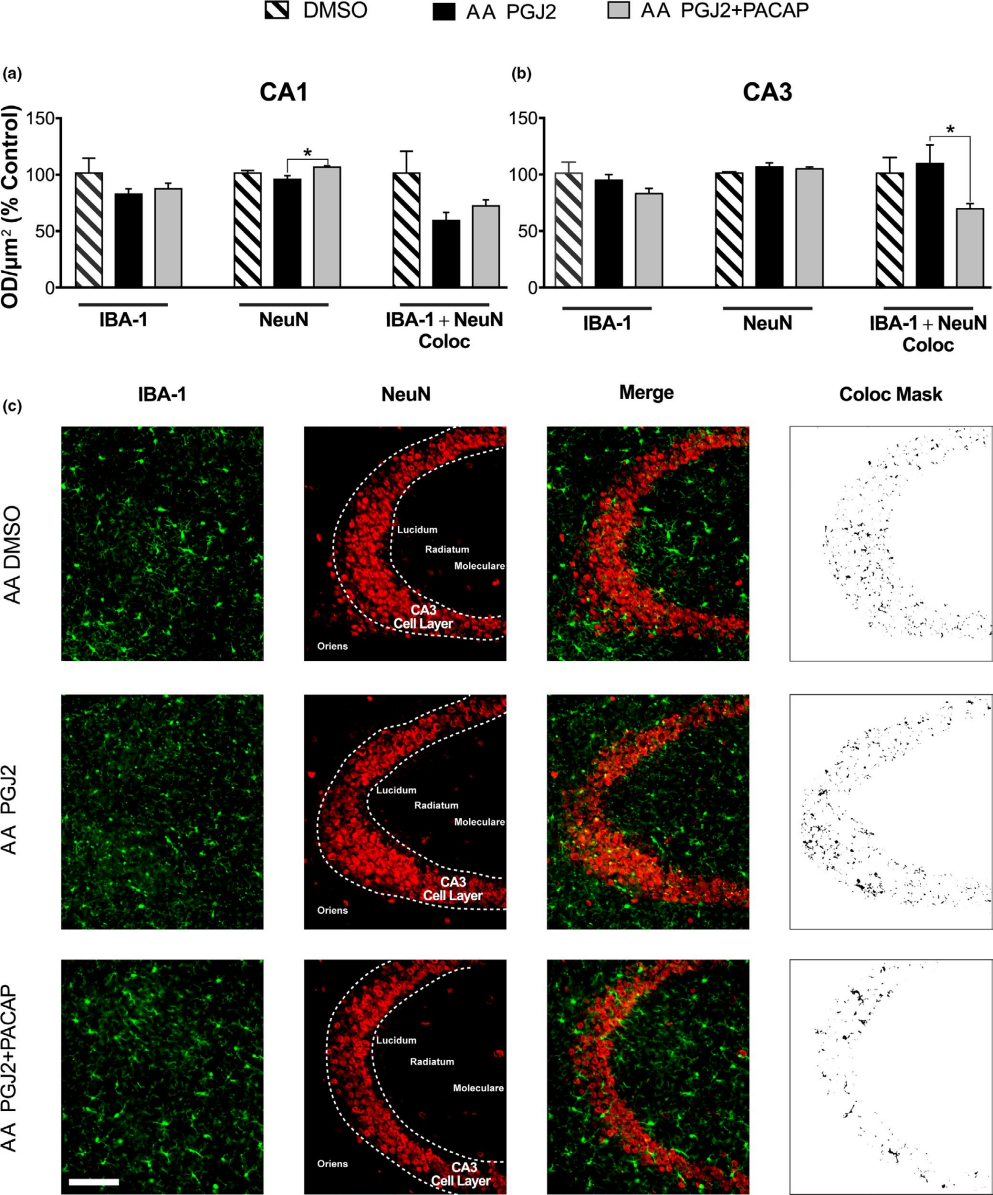
PGJ2‐treated AA show an increase in CA3 microglial levels that is mitigated by PACAP27. Iba‐1, NeuN, and colocalization analyses in (a) CA1 and (b) CA3 for AA treated with PGJ2 versus PGJ2 + PACAP27. (a) In CA1, Iba‐1 expression is not significant; NeuN expression is significant (*g = 2.38); Iba‐1 + NeuN colocalization is not significant. (b) In CA3, Iba‐1 and NeuN are not significant; Iba‐1 + NeuN colocalization is significant (*g = 1.33) (c) IHC for Iba‐1 (green) and NeuN (red) and colocalization (yellow). Stippled area shows CA3 cell layer; Coloc Mask represents the ImageJ mask of CA3 cell layer used in the analysis; bar = 100 µm

**Figure 6:**
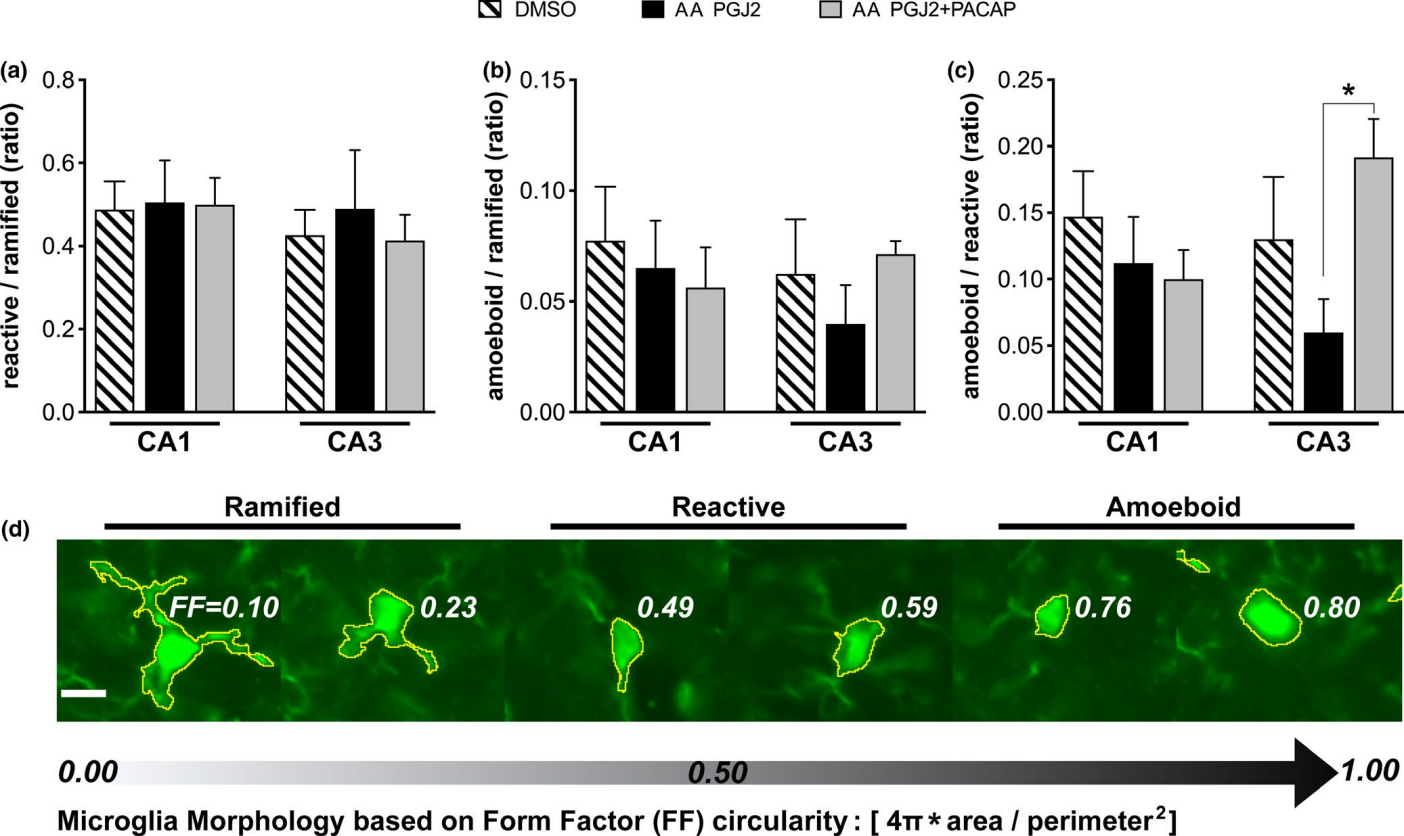
PGJ2‐treated AA show decreased amoeboid:reactive microglia ratio in CA3 that is mitigated by PACAP27. Iba‐1 was used to analyze microglial morphology in hippocampal subregions for AA treated with PGJ2 versus PGJ2 + PACAP27. (a) Reactive/ramified microglia ratios show no significant differences between treatments. (b) Amoeboid/ramified microglia ratios show no significant differences between treatments. (c) Amoeboid/reactive microglia ratios show PGJ2‐treated AA mice exhibited significantly decreased ratios compared with PGJ2 + PACAP‐treated AA mice (d) Images represent microglia form factor (FF) analysis based on circularity of detected IBA‐1‐positive signal. Yellow selection highlights detected microglia particles; bar = 10 µm

All data are expressed as the average ± *SEM*. Statistical analyses were performed with GraphPad Prism 6 (GraphPad Software) or SPSS 22 (IBM Corp.). Parametric analyses were chosen based on previous experiments demonstrating a linear relationship between increasing doses of PGJ2 delivered into the brain and increasing levels of neurotoxicity and IBA‐1 expression (Shivers et al., 2014). Post hoc comparisons utilized Bonferroni's and Dunnett's corrections, as needed per comparison, to balance the probability of committing Type I and II errors (Lee & Lee, 2018). Three‐way repeated measures ANOVAs were used to evaluate behavioral effects across training days (Figure 2). Two‐way ANOVAs were used to analyze retention performance across AA and YA (Figure 3a,c,e), and *t* tests were used to analyze AA PGJ2 versus AA PGJ2 + PACAP (Figure 3b,d,f). One‐way ANOVAs were used to evaluate immunohistochemistry optical density within each brain region followed by controlled comparisons between drug groups (Figures 4, 5, 6). Bonferroni‐corrected *t* tests were used for post hoc analyses. Microglia morphology ratios were analyzed with a Welch's corrected ANOVA and Dunnett's T3 corrected *t* tests, as quantification revealed that across microglia classes, homogeneity of variance was not preserved (see section 3.5). In behavioral and immunohistochemistry data, partial eta squared ($\eta_{p}^{2}$; for ANOVA designs) and Hedge's unbiased g (*g*; for univariate post hoc and *t* test comparisons) were used to calculate effect sizes for results, using Microsoft Excel^®^ as previously described (Avila et al., 2018). Effect sizes were deemed appropriate to account for potential low power of hypothesis tests. The thresholds for large effect sizes in our studies were as follows: when $\eta_{p}^{2}$ was 0.2 or higher, and when *g* was 0.8 or higher. Effect sizes below these criteria were deemed moderate. A *p*‐value <.05 and large effect sizes were considered statistically significant.

### 2.9 Ethics approval and consent to participate

All experimental conditions, housing, and drug administration procedures were reviewed and approved by the Institutional Animal Care and Use Committee (IACUC) of Hunter College.

## 3 RESULTS

### 3.1 Effects of PGJ2 on spatial learning in aged adult (AA) versus young adult (YA)

In order to evaluate CA1 hippocampal function, we use the RAM with four baited and four unbaited arms that allows for both % correct scores and specific error types including spatial reference and working memory errors to be analyzed (Figure 1b). A three‐way ANOVA of RAM performance across 12 training days, 3 drug treatments, and 2 ages revealed an overall significant effect of days ($\eta_{p}^{2}$ = 0.51, F_11, 253_ = 23.441, *p* < .001) and an overall significant interaction effect of age by drug ($\eta_{p}^{2}$ = 0.24, F_1, 23_ = 7.179, *p* = .013). Bonferroni‐corrected post hoc comparisons revealed significant interaction effects of age and drug on day 11 ($\eta_{p}^{2}$ = 0.16, F_1, 23_ = 4.396, *p* < .05) and day 12 ($\eta_{p}^{2}$ = 0.21, F_1, 23_ = 6.207, *p* < .05). Bonferroni pairwise comparisons between drug treatments across training days drugs revealed significant effects on day 1 ($\eta_{p}^{2}$ = 0.47, F_2, 23_ = 10.101, *p* < .01; PGJ2 + PACAP versus DMSO, *p* < .01; PGJ2 + PACAP versus PGJ2, *p* < .05) and day 7 ($\eta_{p}^{2}$ = 0.31, F_2, 23_ = 5.064, *p* < .05; PGJ2 versus DMSO, *p* < .05; PGJ2 versus PGJ2 + PACAP, *p* < .05). Bonferroni pairwise comparisons between ages across training days revealed a significant effect on day 1 ($\eta_{p}^{2}$ = 0.27, F_1, 23_ = 8.398, *p* < .01). Independent two‐way ANOVA comparisons were also produced to more closely analyze our data. Our results show that DMSO‐treated AA and YA show equivalent RAM performance. DMSO‐treated AA versus YA show an effect of training across days representing spatial learning ($\eta_{p}^{2}$ = 0.4, F_11, 88_ = 5.254, *p* < .01), with no effect of age or post hoc differences (Figure 2a). PGJ2‐treated AA versus YA show an effect of training across days ($\eta_{p}^{2}$ = 0.67, F_11,110_ = 20.01, *p* < .01), age ($\eta_{p}^{2}$ = 0.81, F_1,10_ = 42.56, *p* < .01), and an interaction ($\eta_{p}^{2}$ = 0.26, F_11,110_ = 3.59, *p* < .01). Post hoc analyses show differences on d1 (*g* = 2.4, t_120_ = 4.392, **p < .01), d5 (*g* = 2.2, t_120_ = 3.456, **p < .01), d8 (*g* = 1.8, t_120_ = 3.903, **p < .01), d9 (*g* = 1.4, t_120_ = 3.176, *p < .05), d11 (*g* = 2.3, t_120_ = 5.199, **p < .01), and d12 (*g* = 2.2, t_120_ = 4.795, **p < .01) (Figure 2b). PGJ2 + PACAP27 versus PGJ2 treatment in AA shows an effect of training across days ($\eta_{p}^{2}$ = 0.56, F_11,121_ = 14.06, *p* < .01) and an effect of drug treatment ($\eta_{p}^{2}$ = 0.46, F_1,11_ = 9.422, *p* < .05). Post hoc analyses show differences on training day 9 (t_132_ = 3.049, *p < .05; Figure 2c). Additional analyses for % correct scores between PGJ2‐ versus DMSO‐treated YA show an effect of training across days ($\eta_{p}^{2}$ = 0.69, F_11, 66_ = 13.31, *p* < .01) but no effect of drug treatment. Conversely, PGJ2‐ versus DMSO‐treated AA show an effect of training across days ($\eta_{p}^{2}$ = 0.43, F_11,132_ = 8.928, *p* < .01) and an effect of drug treatment ($\eta_{p}^{2}$ = 0.41, F_1,12_ = 8.395, *p* < .05). % correct scores for DMSO‐ versus PGJ2 + PACAP27‐treated AA show an effect of training across days ($\eta_{p}^{2}$ = 0.48, F_11,165_ = 13.72, *p* < .01) with no effect of drug treatment. These results show that PGJ2‐treated AA have reduced spatial learning performance compared to PGJ2‐treated YA and PGJ2 + PACAP27‐treated AA, while YA treated with PGJ2 have intact spatial learning.

A three‐way ANOVA of RAM working memory errors (WME) across 12 training days, 3 drug treatments, and 2 ages revealed an overall significant effect of days ($\eta_{p}^{2}$ = 0.77, F_11, 13_ = 4.028, *p* < .05). Bonferroni‐corrected post hoc comparisons revealed significant overall drug effects on day 1 ($\eta_{p}^{2}$ = 0.35, F_2, 23_ = 6.25, *p* < .01) and day 7 ($\eta_{p}^{2}$ = 0.3, F_2, 23_ = 5.045, *p* < .05). Bonferroni pairwise comparisons between drug treatments across training days revealed an overall effect on day 1 ($\eta_{p}^{2}$ = 0.32, F_2,23_ = 5.436, *p* < .05; DMSO versus PGJ2 + PACAP, *p* < .01; separately PGJ2 versus PGJ2 + PACAP, *p* < .05) and day 7 ($\eta_{p}^{2}$ = 0.27, F_2,23_ = 4.354, *p* < .05; DMSO versus PGJ2, *p* < .05). Pairwise comparison across ages revealed a significant overall effect on day 10 ($\eta_{p}^{2}$ = 0.17, F_1, 23_ = 4.552, *p* < .05). Independent two‐way ANOVA comparisons were also produced to more closely analyze our data. WME between YA versus AA treated with DMSO show an effect of training across days ($\eta_{p}^{2}$ = 0.21, F_11, 88_ = 2.135, *p* < .05; Figure 2d) but no effect of age. Similarly, YA versus AA treated with PGJ2 show an effect of training across days ($\eta_{p}^{2}$ = 0.24, F_11, 110_ = 3.241, *p* < .01; Figure 2e) but no effect of age. WME between PGJ2 versus PGJ2 + PACAP27 treated AA did not show an effect of drug treatment or training across days (Figure 2f). Additional WME analyses between PGJ2 versus DMSO in AA treated show an effect of training across days ($\eta_{p}^{2}$ = 0.22, F_11,132_ = 3.482, *p* < .01) but no effect of drug treatment. Together these results indicate that PGJ2‐induced learning deficits in % correct scores are not driven by WME. WME between DMSO versus PGJ2 + PACAP27 in AA treated show an effect of training ($\eta_{p}^{2}$ = 0.2, F_11,121_ = 2.824, *p* < .01) and an effect of drug treatment ($\eta_{p}^{2}$ = 0.36, F_1, 11_ = 6.256, *p* < .05) indicating that PACAP27 treatment does not mitigate PGJ2‐induced WME deficits.

A three‐way ANOVA of RAM reference memory errors (RME) across 12 training days, 3 drug treatments, and 2 ages revealed an overall significant effect of days ($\eta_{p}^{2}$ = 0.96, F_11, 13_ = 31.16, *p* < .001). Bonferroni‐corrected post hoc comparisons revealed an overall effect of age on day 1 ($\eta_{p}^{2}$ = 0.24, F_1, 23_ = 7.36, *p* < .05), an overall effect of drug on day 3 ($\eta_{p}^{2}$ = 0.28, F_2, 23_ = 4.556, *p* < .05), and overall interaction effect of drug on day 12 ($\eta_{p}^{2}$ = 0.21, F_1, 23_ = 6.185, *p* < .05). Bonferroni pairwise comparisons between drug treatments across training days revealed an overall effect on day 3 ($\eta_{p}^{2}$ = 0.28, F_2, 23_ = 4.573, *p* < .05; DMSO versus PGJ2 *p* = .01). Bonferroni pairwise comparisons between ages across training days revealed an overall effect on day 1 ($\eta_{p}^{2}$ = 0.27, F_1, 23_ = 8.457, *p* < .01). Independent two‐way ANOVA comparisons were also produced to more closely analyze our data. RME between YA and AA treated with DMSO show an effect of training across days ($\eta_{p}^{2}$ = 0.46, F_11, 88_ = 6.875, *p* < .01; Figure 2g) but not effect of age. PGJ2‐treated YA versus AA show an effect of age ($\eta_{p}^{2}$ = 0.95, F_11, 10_ = 16.65, *p* < .01), training across days ($\eta_{p}^{2}$ = 0.72, F_11, 110_ = 25.71, *p* < .01), and an interaction ($\eta_{p}^{2}$ = 0.21, F_11, 110_ = 2.643, *p* < .01; Figure 2h). Post hoc analyses show differences on training days 1, 11, and 12 (d1 *g* = 1.8, t_120_ = 3.04, *p < .05; d11 *g* = 1.9, t_120_ = 3.512, **p < .01; d12 *g* = 2.8, t_120_ = 4.457, **p < .01). PGJ2‐ versus PGJ2 + PACAP27‐treated AA show an effect of training across days ($\eta_{p}^{2}$ = 0.58, F_11, 121_ = 15.16, *p* < .01; Figure 2i) but no effect of drug treatment. Additional analyses for RME between PGJ2‐ versus DMSO‐treated AA show an effect of training ($\eta_{p}^{2}$ = 0.56, F_11, 132_ = 15.5, *p* < .01) and drug treatment ($\eta_{p}^{2}$ = 0.34, F_1, 12_ = 6.216, *p* < .05). DMSO‐ versus PGJ2 + PACAP27‐treated AA show an effect of training across days ($\eta_{p}^{2}$ = 0.55, F_11, 121_ = 13.65, *p* < .001), but no effect of drug treatment. These results indicate increased reference memory errors in PGJ2‐treated AA but not YA, an effect that is mitigated with PGJ2 + PACAP27.

### 3.2 Effects of PGJ2 on long‐term spatial memory retention in aged adult (AA) versus young adult (YA)

Six days after the last RAM training trial, all mice were given 6 RAM trials with the same sequence of baited/unbaited arms used during acquisition training. A two‐way ANOVA of average % correct scores between YA versus AA treated with PGJ2 versus DMSO shows an effect of age ($\eta_{p}^{2}$ = 0.28, F_1,18_ = 7.039, *p* = .01; Figure 3a), but no interaction of age and drug treatment. Post hoc analyses show differences between PGJ2‐treated YA versus AA (*g* = 2.7, t_18_ = 3.661, **p < .01). A between‐subject's analysis of average performance between AA treated with PGJ2 versus PGJ2 + PACAP27 shows no effect of drug (Figure 3b). A two‐way ANOVA of WME between YA versus AA treated with PGJ2 versus DMSO shows no effect of drug treatment or age (Figure 3c). A between‐subject's analysis of average WME between AA treated with PGJ2 versus PGJ2 + PACAP27 revealed no effect of drug (Figure 3d). A two‐way ANOVA of RME between YA versus AA treated with PGJ2 versus DMSO show an effect of age ($\eta_{p}^{2}$ = 0.38, F_1,18_ = 11.01, *p* < .01) and an interaction ($\eta_{p}^{2}$ = 0.25, F_1,18_ = 6.147 *p* < .05; Figure 3e). Post hoc analyses show differences in PGJ2‐treated YA versus AA (*g* = 2.4, t_18_ = 4.481, **p < .01) and between AA treated with DMSO versus PGJ2 (*g* = 1.39, t_18_ = 3.306, *p < .05). A between‐subject's analysis of average RME between AA treated with PGJ2 versus PGJ2 + PACAP27 is significant (*g* = 1.6, t_11_ = 3.041, *p < .05; Figure 3f). These results show that PGJ2 induces long‐term memory retrieval deficits in AA reflected in % correct scores and RME and PACAP27 mitigates RME deficits but not deficits in % correct.

### 3.3 Effects of PGJ2 on CA1 and CA3 Fluoro‐Jade C staining in the aged adult (AA) versus young adult (YA) hippocampus

A one‐way ANOVA of FJC staining in CA1 showed a significant effect across treatments ($\eta_{p}^{2}$ = 0.501, F_3,20_ = 6.712, *p* < .01; Figure 4a). Post hoc analyses with controlled comparisons show significant differences between AA treated with PGJ2 versus PGJ2 + PACAP27 (*g = 1.63, t_20_ = 3.396, *p* < .01) as well as YA treated with PGJ2 versus AA treated with PGJ2 + PACAP27 (**g = 2.61, t_20_ = 4.236, *p* < .01). PGJ2‐treated AA versus YA are not significant. Post hoc comparisons between DMSO and the remaining groups in CA1 did not reveal significant differences. Analysis of FJC staining in CA3 showed a significant effect size and difference across treatments ($\eta_{p}^{2}$ = 0.81, F_3,20_ = 27.51, *p* < .001; Figure 4b). Post hoc analyses with controlled comparisons show significant differences between PGJ2‐treated YA versus AA (**g = 3.09, t_14_ = 6.702, *p* < .001) and between AA treated with PGJ2 + PACAP27 versus PGJ2 (**g = 3.23, t_20_ = 6.909, *p* < .001). Post hoc comparisons between DMSO and the remaining groups in CA3 revealed significant differences between DMSO and YA PGJ2 (^*g* = 3.17, t_20_ = 5.871, *p* < .001) as well as DMSO and AA PGJ2 + PACAP (!*g* = 3.285, t_20_ = 6.078, *p* < .001). These results indicate that CA3 pyramidal cells have higher levels of neurodegeneration in PGJ2‐treated AA versus YA and this effect is mitigated by PACAP27.

### 3.4 Effects of PGJ2 on Iba‐1 expression in the aged adult (AA)hippocampus

Optical density analyses of Iba‐1, NeuN, and colocalization were carried out between DMSO, AA treated with PGJ2 and PGJ2 + PACAP27 in CA1 and CA3 (Figure 5). In CA1, Iba‐1 expression is not different between groups ($\eta_{p}^{2}$ = 0.201, F_2, 9_ = 1.135, *p* = .364) (Figure 5a). NeuN staining analysis shows a significant effect of group ($\eta_{p}^{2}$ = 0.58, F_2, 9_ = 6.216, *p* < .05). Post hoc comparisons reveal an increase of NeuN in AA treated with PGJ2 + PACAP27 versus PGJ2 (*g = 2.38; t_9_ = 3.497, *p* < .01). Iba‐1 + NeuN colocalization is not different across groups in CA1 ($\eta_{p}^{2}$ = 0.38, F_2, 9_ = 2.81, *p *= .113). Post hoc comparisons between DMSO and the remaining groups in CA1 did not reveal significant differences. In CA3 (Figure 5b), Iba‐1 ($\eta_{p}^{2}$ = 0.202, F_2, 14_ = 1.776; *p* = .205) and NeuN ($\eta_{p}^{2}$ = 0.19, F_2, 14_ = 1.667, *p* = .224) expression are not different between groups. Iba‐1 + NeuN colocalization analysis shows a significant effect size across groups ($\eta_{p}^{2}$ = 0.277, F_2, 13_ = 2.49, *p* = .121), and post hoc analyses reveal a significant decrease in AA treated with PGJ2 + PACAP27 versus PGJ2 (*g = 1.33, t_13_ = 2.107, *p* = .055). Post hoc comparisons between DMSO and the remaining groups in CA3 did not reveal significant differences. These results indicate increased PGJ2‐induced vulnerability to chronic neural inflammation and spread of inflammatory activity specifically in AA.

### 3.5 Effects of PGJ2 on Iba‐1 microglial morphology in the aged adult (AA) hippocampus

Morphological analyses of microglia were carried out between DMSO, AA treated with PGJ2 and PGJ2 + PACAP27 in CA1 and CA3. A two‐way ANOVA of microglia counts in the whole hippocampus between groups and across shape class revealed an overall difference between shape classes ($\eta_{p}^{2}$ = 0.82, F_2,42_ = 90.4, *p* < .001), no difference between treatments ($\eta_{p}^{2}$ = 0.18, F_2,42_ = 0.389, *p* = .68), and no interaction of shape class and treatment ($\eta_{p}^{2}$ = 0.16, F_4,42_ = 0.171, *p* = .95). A Levene statistic revealed that across groups, homogeneity of variance was not preserved ($\eta_{p}^{2}$ = 0.34, F_8,42_ = 2.68, *p* < .05) in these data. These results indicated that further analyses should utilize corrected statistics (Welch's ANOVA and Dunnett's T3) to account for the unequal variances and differences between shape classes. Ratios of counts were used to analyze the proportional levels of each microglia class relative to each other, a measure that can characterize inflammatory responses better compared to raw counts (Corwin et al., 2018). The ratios of reactive to ramified microglia revealed no significant differences between groups in neither CA1 (W_2,8.6_ = 0.01, *p* = .98) nor CA3 (W_2,8.1_ = 0.11, *p* = .89) with one‐way Welch's ANOVAs (Figure 6a). The ratios of amoeboid to ramified microglia revealed no significant differences between groups in neither CA1 (W_2,9.07_ = 0.22, *p* = .80) nor CA3 (W_2,6.8_ = 1.35, *p* = .319) with one‐way Welch's ANOVAs (Figure 6b). The ratios of amoeboid to ramified microglia revealed no significant differences between groups in CA1 (W_2,8.56_ = 0.62, *p* = .55) and a significant difference between groups in CA3 (W_2,9.1_ = 5.647, *p* < .05) with one‐way Welch's ANOVAs (Figure 6c). Post hoc Dunnett's corrected controlled comparisons revealed a significant difference between AA PGJ2 and AA PGJ2 + PACAP27 (*t_8.99_ = 3.47, *p* < .05). Post hoc comparisons between DMSO and the remaining groups in CA1 and CA3 did not reveal significant differences.

## 4 DISCUSSION

Our studies demonstrate that stereotaxic infusion of the cyclooxygenase product of inflammation PGJ2 into the CA1 region of the hippocampus induces age‐dependent cognitive deficits and neurodegeneration that spreads retrogradely to the CA3 region. We focused our investigation on the product of inflammation PGJ2 because it is highly neurotoxic (Li, Jansen, et al., 2004), and its levels were shown in rodents to increase upon brain injuries such as stroke and TBI (Hickey et al., 2007; Kunz et al., 2002), which are risk factors for AD, reviewed in Figueiredo‐Pereira et al. (2015).

### 4.1 PGJ2 induces spatial learning and memory deficits in aged adult (AA) but not young adult (YA)

Our behavioral assessments show that PGJ2‐treated AA mice have decreased RAM spatial learning compared to YA and compared to AA treated with DMSO. We use the RAM to characterize hippocampal function as this task allows to identify both reference and working memory systems together. Previous studies characterizing hippocampal function following injury evaluated these two memory systems independently, most commonly using the Morris water maze (Bramlett, Green, & Dietrich, 1997; Dash, Moore, & Dixon, 1995; Scheff, Baldwin, Brown, & Kraemer, 1997; Smith, Okiyama, Thomas, Claussen, & McIntosh, 1991). In order to identify whether the brain can compensate for deficits in one memory system with another, it is important to evaluate these systems in a single paradigm (Lyeth et al., 1990; Soblosky et al., 1996). Learning the RAM long‐term rules involving the location of the baited arms (reference memory) while simultaneously remembering which arms have been visited (working memory) is particularly challenging. The RAM task, utilizing four baited and four unbaited arms, taxes a rodent's ability to juggle both short‐term working and long‐term reference memory systems (Abrahams, Pickering, Polkey, & Morris, 1997; Baddeley, 1981). In addition to its ethological relevance to rodent foraging behaviors (Floresco, Seamans, & Phillips, 1997), the RAM requires the hippocampus (Jarrard, 1978; Olton & Papas, 1979) and prefrontal cortex (Floresco et al., 1997) for learning the task.

In order to evaluate the long‐term nature of the PGJ2‐induced learning deficits, we use a massed training protocol for RAM. The effects of extensive training have been reported to mitigate learning and memory deficits across various paradigms (Beatty, Bierley, & Boyd, 1985; Daumas et al., 2008; Wallace, Krauter, & Campbell, 1980). Several studies have shown a reversal of short‐term memory deficits in over trained transgenic mice and an elimination of the spatial memory deficits with subsequent retraining (Daumas et al., 2008; Zhang, Storm, & Wang, 2011). Additional evidence has demonstrated that over training or training to criteria minimizes memory deficits in older animals (Beatty et al., 1985; Stewart, Mitchell, & Kalant, 1989; Wallace et al., 1980). Similarly, we use a robust training protocol on the RAM that is known to result in successful memory retrieval up to one month post‐training in male rats (Sebastian, Vergel, et al., 2013). This robust training protocol could also explain the lack of differences in spatial learning and memory performance between DMSO‐treated AA and YA as numerous studies have shown the hippocampus (HPC) to be an early target of age‐related structural and physiological changes that may contribute to learning and memory deficits (Driscoll & Sutherland, 2005; Rosenzweig & Barnes, 2003; Stephens et al., 2011). Based on these studies, the robust nature of the RAM training protocol used suggests that the deficits in spatial learning in PGJ2‐treated AA are resistant to overtraining and highlights the importance for CA3 in spatial learning.

Our results show that PGJ2‐treated AA have deficits in spatial memory retrieval that is reflected in their RAM % correct scores and RME. These results highlight the persistent nature of the PGJ2‐induced memory deficit. In contrast to the learning deficits produced by PGJ2 in AA (Figure 2), the retention deficit in the same group (Figure 3) reveals how PGJ2 in AA can affect the long‐term memory capacities in these mice. Several reports have shown that neurochemistry and cellular morphology associated with brain injury can change remarkably during an acute recovery phase (Chen, Pickard, & Harris, 2003; Hoskison et al., 2009; Newcomb, Zhao, Pike, & Hayes, 1999). These findings are relevant to the data we present here, which identify the persistent nature of the PGJ2‐induced memory deficit and highlight the brain's inability to compensate for the deficits with experience as shown in many studies. It remains to be determined whether combined interventions will be valuable alternative behavioral therapies for improving long‐lasting PGJ2‐induced deficits involving diet and exercise (Chen et al., 2013; Piao et al., 2013) and environmental enrichment (Hoffman et al., 2008; Matter, Folweiler, Curatolo, & Kline, 2011; Monaco et al., 2013).

### 4.2 PGJ2 induces a retrograde spread of neurodegeneration as well as microglial activation in aged adults (AA) but not young adults (YA)

We quantified FJC staining to evaluate the neurotoxicity induced by PGJ2 microinjected into the CA1 subfield. FJC has been found to stain actively degenerating neurons and terminals (Schmued et al., 2005). Our results show no differences between PGJ2‐treated AA compared to YA in CA1 which suggests that multiple injections within this subfield produce equivalent and persistent neurodegeneration in this region independent of age. However, in CA3, PGJ2‐treated AA show higher levels of FJC staining, compared to YA PGJ2 mice, that is mitigated with PACAP27. Furthermore, DMSO FJC levels are comparable to AA PGJ2, suggesting that the increased levels of FJC in AA PGJ2 are qualitatively dissimilar to DMSO and may include activated microglia (Damjanac et al., 2007). This suggests that PGJ2‐induced neurotoxicity is associated with both microglial aviation and FJC staining. Alternatively, this result indicates that AA mice treated with PACAP27, similar to YA PGJ2, may have not undergone any neurodegeneration in CA3, unlike DMSO‐ or PGJ2‐treated AA mice. This suggests that AA mice perpetuate a spread of PGJ2‐induced neuroinflammation that initiates in CA1, and that PACAP27 or YA mice prevent this spread of damage. The retrograde spread of PGJ2‐induced neurotoxicity from CA1 to CA3 is consistent with studies showing that prostaglandins can spread between cells via exosomes. Exosomes containing prostaglandins, including PGJ2, are extracellular bioactive vesicles that can be internalized by neighboring cells (Subra et al., 2010) and taken up at synapses in an activity‐dependent manner regulated by calcium (Chivet et al., 2013; Lachenal et al., 2011; Morel et al., 2013; Perez‐Gonzalez, Gauthier, Kumar, & Levy, 2012). Reports have suggested that exosomes containing prostaglandins may also play a role in the spread of pathology across a variety of neurodegenerative disorders (Schneider & Simons, 2013). Our results with FJC reveal a PGJ2‐dependent spread of pathology across two subregions of the aged hippocampus, but further experiments are needed to determine its precise mechanism.

Alternatively, these results could also indicate a diffusion of the drug from the area of delivery, CA1, to the adjacent CA3 region. It is unclear how PGJ2 delivery or upregulation in specific brain regions can affect or be transported to adjacent regions. However, an understanding of exosome transport of prostaglandins could provide a framework to examine this phenomenon (Subra et al., 2010). Future studies should seek to elucidate mechanisms responsible for the diffusion of PGJ2 in the hippocampus from one region to another.

To characterize the role of microglia in PGJ2‐induced hippocampal‐dependent memory deficits, we conducted further immunohistochemistry analyses using Iba‐1. Quantification of microglial expression using Iba‐1 staining identifies enhanced expression within CA3 pyramidal neuron layer in PGJ2‐treated AA compared to AA treated with PGJ2 + PACAP27. It is well known that PGJ2 can exacerbate neuroinflammation, reviewed in Figueiredo‐Pereira et al. (2015). However, the mechanisms by which PGJ2 induces microglia activation are still unknown, as well as its contribution to microglia‐induced pathology in AD. Studies of microglial markers in postmortem brains indicate that Iba‐1 expression increases in AD brains compared to controls, contributing to microglial activation (Hopperton, Mohammad, Trépanier, Giuliano, & Bazinet, 2017). Our previous studies with neuronal cells show that PGJ2 increases the levels of the pro‐inflammatory cytokine interleukin‐1 (IL‐1) (Li, Melandri, et al., 2004). Given that IL‐1 mediates microglia activation in LPS‐injected mice (Tanaka et al., 2013), we hypothesize that PGJ2 induces microglia either directly or indirectly through IL‐1 activation. Increases in IL‐1 levels are relevant to neurodegenerative disorders as shown in PD pathogenesis, where IL‐1 levels are high in patients (Mogi et al., 1994), and in animal models of PD (Koprich, Reske‐Nielsen, Mithal, & Isacson, 2008; Pott Godoy, Tarelli, Ferrari, Sarchi, & Pitossi, 2008) and animal models of AD (Ghosh et al., 2013; Kitazawa et al., 2011). Further studies are needed to clarify this mechanism of activation.

Microglia are able to modulate the brain's activity and regulate protein turnover via phagocytic activity (Kettenmann, Kirchhoff, & Verkhratsky, 2013; Sierra et al., 2010). Functional Iba‐1‐dependent microglial phagocytosis of amyloid beta has also reported (Krabbe et al., 2013), suggesting a role for microglia in function in the onset and progression of neurodegenerative diseases. Further studies have found that microglia can phagocytose tau aggregates in neurons (Asai et al., 2015; Brelstaff, Tolkovsky, Ghetti, Goedert, & Spillantini, 2018; Luo et al., 2015), and our previous work has shown that PGJ2 can produce calpain‐dependent tau cleavage and produce ubiquitin aggregates (Figueiredo‐Pereira et al., 2015; Kiprowska et al., 2017; Metcalfe et al., 2012). These studies suggest that PGJ2 infusion into the hippocampus could be increasing microglial‐dependent phagocytosis of neuronal debris or degenerating neuronal processes containing tau aggregates. Microglial‐dependent phagocytic activity on neurons has been shown to alter neuronal spine morphology (Weinhard et al., 2018), an event that may trigger long‐term synaptic depression via AMPA‐receptor endocytosis (Zhang et al., 2014). Our data do not show significant changes in overall levels of IBA‐1 microglia, suggesting that microglial recruitment is not significantly altered as a result of PGJ2‐induced neuroinflammation. This notion is supported by our recent studies (Corwin et al., 2018) showing that PGJ2 did not significantly change the overall numbers of activated microglia in the substantia nigra of PGJ2‐infused rats. However, PGJ2 induced a significant rise in phagocytic microglia in the PGJ2‐treated rats. Our current study highlights microglial responses four (4) weeks after the end of infusions, thus allowing sufficient time for a complete neuroinflammatory response to take place, that may have included macrophage infiltration from the periphery (Mildner et al., 2007; Sevenich, 2018) as well as microglial migration from neighboring brain regions (Carbonell, Murase, Horwitz, & Mandell, 2005; Zhang, Nance, Alnasser, Kannan, & Kannan, 2016). Nonetheless, our data confirm a shift in ratios of amoeboid to reactive microglia in CA3, with AA PGJ2 showing a decreased ratio compared to AA PGJ2 + PACAP27. This suggests that PGJ2 prevents a resolution of inflammation, as amoeboid microglia have been linked to phagocytic activity (Kettenmann, Hanisch, Noda, & Verkhratsky, 2011). These changes in microglia ratios and colocalization with neurons in CA3 suggest that PGJ2 infusion into CA1 induces differential susceptibilities in the CA3 subregion that affect learning and memory.

### 4.3 PACAP27 mitigates PGJ2‐induced memory deficits and neurotoxicity in aged adult (AA)

We also showed that the neuroprotective peptide PACAP27 prevented the PGJ2‐induced spatial learning and memory deficits in AA. While we did not measure endogenous PACAP levels in our studies, we hypothesize that the ability for the enhanced performance and reduced CA3 microglia expression in PGJ2 + PACAP27‐treated AA compared to PGJ2‐treated AA is driven, in part by the internalized PACAP27 that functions to remediate the actions of PGJ2. This is consistent with studies showing that PACAP levels are reduced in aged mice and nonhuman primates (Han et al., 2017) and that PACAP is involved in synaptic plasticity and memory (MacDonald, Jackson, & Beazely, 2007; Roberto, Scuri, & Brunelli, 2001) as well as in stress‐related behavioral responses (Hammack et al., 2009; Tsukiyama et al., 2011). Together these studies support PACAP as a potential therapeutic for remediating age‐dependent cognitive decline. PACAP can be neuroprotective against β‐amyloid‐induced mitochondrial function (Han et al., 2014), which is also triggered by PGJ2, leading to increases in oxidative stress and apoptosis (Kondo, Oya‐Ito, Kumagai, Osawa, & Uchida, 2001; Lee, Kwon, Park, Kim, & Woo, 2009; Paulitschke et al., 2012). Interestingly, PACAP levels are reduced in humans with AD which is correlated with both cognitive decline and severity of pathologic marker expression (Han et al., 2015). Based on these studies, we presume that PACAP27 may be in part reducing PGJ2‐induced mitochondrial impairment. Further studies are needed to confirm this mechanism.

PACAP also exerts neurotrophic and neuroprotective activities (Reglodi et al., 2012), promoting neurite outgrowth (Ogata et al., 2015), neuroprotection from ischemia (Ohtaki et al., 2006), and increases in hippocampal neurogenesis following environmental enrichment (Ago et al., 2011). Additionally, PACAP exerts potent anti‐inflammatory properties inducing decreases in pro‐inflammatory mediators interleukin IL‐12, tumor necrosis factor TNF‐α, and nitric oxide and induces anti‐inflammatory cytokine IL‐10 in LPS stimulated macrophages (Delgado & Ganea, 1999; Delgado, Munoz‐Elias, Gomariz, & Ganea, 1999). Whether PACAP acts directly by reducing apoptotic neuronal death (Ohtaki, Nakamachi, Dohi, & Shioda, 2008) or indirectly via modulation of the inflammatory processes (Dejda et al., 2011; Ohtaki et al., 2006), PACAP can act as a potent regulator of the microglial response in vivo after stroke (Brifault et al., 2015) and TBI, by inhibiting TLR4/MyD88/NF‐κB signaling in microglia and neurons, reducing neuronal death (Mao et al., 2012). These studies offer a potential neural mechanism for the ability of PACAP to mitigate PGJ2‐induced spatial memory deficits in AA. Several studies have shown that increased microglial expression reduces hippocampal‐dependent memory performance (Hou et al., 2014; Morris et al., 2013; Smith et al., 2018). In fact, overactivation of microglia plays a pivotal role in neuroinflammation and in neurodegenerative disorders leading to cognitive decline. It has been proposed that chronic inflammation involving PGJ2 creates a self‐propagating cycle leading to uncontrolled, prolonged inflammation that drives the chronic progression in neurodegenerative diseases [reviewed in (Figueiredo‐Pereira et al., 2015; Gao & Hong, 2008)]. Thus, our data support that increasing cAMP levels with PACAP27 may be an efficient age‐dependent therapeutic approach to prevent/reduce inflammation, neurodegeneration, microglia expression and their associated memory deficits. PACAP peptides could offer an alternative approach to phosphodiesterase inhibitors being tested against age‐related and AD cognitive decline (Heckman, Blokland, & Prickaerts, 2017; Yang et al., 2015).

### 4.4 Conclusions and clinical implications

In conclusion, our data showing that PGJ2 leads to age‐dependent neurodegeneration underscore the relevance of prostaglandin signaling as a modulator of the long‐term effects of neuroinflammation. Targeting neurotoxic factors downstream of cyclooxygenases, such as PGJ2, offer great promise as a new therapeutic strategy that would not alter the homeostatic balance maintained by cyclooxygenases. Prostaglandin D2/J2 signaling offers a range of practical therapeutic targets, including prostaglandin synthase (L‐PGDS), dehydrogenase (15‐PGDH) a prostaglandin‐degrading enzyme, receptors (DP2, membrane; PPARγ, nuclear), and covalent protein modification (Michael adducts), which is particular to PGJ2 (Figueiredo‐Pereira, Corwin, & Babich, 2016), reviewed in Figueiredo‐Pereira et al. (2015). Our newly developed PGJ2‐induced mouse model is a novel platform for testing potential neuroprotective drugs against PGJ2‐induced memory deficits.

## CONFLICT OF INTEREST

The authors declare that the research was conducted in the absence of any commercial or financial relationships that could be construed as a potential conflict of interest.

## AUTHOR CONTRIBUTIONS

MEFP, PAS, and PR designed the experiments and wrote the manuscript. MK and TJL planned and carried out the stereotaxic surgeries and drug infusions. JAA conducted the behavioral testing and statistical analysis. JAA and MK planned and carried out the immunohistochemistry staining and statistical analyses. All authors approved the final manuscript for submission.

## Data Availability

The data that support the findings of this study are available from the corresponding author upon reasonable request.
